# Comprehensive scientometrics and visualization study profiles lymphoma metabolism and identifies its significant research signatures

**DOI:** 10.3389/fendo.2023.1266721

**Published:** 2023-09-26

**Authors:** Song-Bin Guo, Dan-Qi Pan, Ning Su, Man-Qian Huang, Zhen-Zhong Zhou, Wei-Juan Huang, Xiao-Peng Tian

**Affiliations:** ^1^ Department of Medical Oncology, Sun Yat-sen University Cancer Center, Guangzhou, China; ^2^ State Key Laboratory of Oncology in South China, Collaborative Innovation Center of Cancer Medicine, Sun Yat-sen University Cancer Center, Guangzhou, China; ^3^ Department of Hematology, The First Affiliated Hospital of Jinan University, Guangzhou, China; ^4^ Department of Oncology, Guangzhou Chest Hospital, Guangzhou, China; ^5^ Department of Radiology, Sun Yat-sen University Cancer Center, Guangzhou, China; ^6^ Department of Pharmacology, College of Pharmacy, Jinan University, Guangzhou, China

**Keywords:** lymphoma metabolism, apoptosis, radiomics, scientometrics, visualization, study strategy

## Abstract

**Background:**

There is a wealth of poorly utilized unstructured data on lymphoma metabolism, and scientometrics and visualization study could serve as a robust tool to address this issue. Hence, it was implemented.

**Methods:**

After strict quality control, numerous data regarding the lymphoma metabolism were mined, quantified, cleaned, fused, and visualized from documents (n = 2925) limited from 2013 to 2022 using R packages, VOSviewer, and GraphPad Prism.

**Results:**

The linear fitting analysis generated functions predicting the annual publication number (y = 31.685x - 63628, R² = 0.93614, Prediction in 2027: 598) and citation number (y = 1363.7x - 2746019, R² = 0.94956, Prediction in 2027: 18201). In the last decade, the most academically performing author, journal, country, and affiliation were Meignan Michel (n = 35), European Journal of Nuclear Medicine and Molecular Imaging (n = 1653), USA (n = 3114), and University of Pennsylvania (n = 86), respectively. The hierarchical clustering based on unsupervised learning further divided research signatures into five clusters, including the basic study cluster (Cluster 1, Total Link Strength [TLS] = 1670, Total Occurrence [TO] = 832) and clinical study cluster (Cluster 3, TLS = 3496, TO = 1328). The timeline distribution indicated that radiomics and artificial intelligence (Cluster 4, Average Publication Year = 2019.39 ± 0.21) is a relatively new research cluster, and more endeavors deserve. Research signature burst and linear regression analysis further confirmed the findings above and revealed additional important results, such as tumor microenvironment (a = 0.6848, R² = 0.5194, p = 0.019) and immunotherapy (a = 1.036, R² = 0.6687, p = 0.004). More interestingly, by performing a “Walktrap” algorithm, the community map indicated that the “apoptosis, metabolism, chemotherapy” (Centrality = 12, Density = 6), “lymphoma, pet/ct, prognosis” (Centrality = 11, Density = 1), and “genotoxicity, mutagenicity” (Centrality = 9, Density = 4) are crucial but still under-explored, illustrating the potentiality of these research signatures in the field of the lymphoma metabolism.

**Conclusion:**

This study comprehensively mines valuable information and offers significant predictions about lymphoma metabolism for its clinical and experimental practice.

## Highlights

1. Our study provides a comprehensive visualization overview of the evolution and linkage of global research on the lymphoma metabolism.
2. By introducing linear regression and statistical analysis, we identify several significant research signatures regarding the lymphoma metabolism.
3. By utilizing the Walktrap algorithm based on the Random Walk strategy, we predict several under-explored areas that are essential for the lymphoma metabolism but still need further study.

## 1 Introduction

Lymphoma is a cluster of malignant hematologic diseases with a complex and highly heterogeneous classification and considerable variability in prognosis and response to treatment (1–6). Over the centuries, multiple treatment modalities have been developed, comprising conventional chemotherapy, radiation therapy, small molecule targeted agents, immunotherapy, and transplantation (2, 4–9). Recently, the efforts of oncology biologists and clinicians yielded considerable essential research advances in this field, and the development of new drugs has been flourishing. Novel agents, such as BTK inhibitors, BCL2 inhibitors, PD-1 monoclonal antibodies, and epigenetic agents, such as Decitabine, Azacitidine, and Cidabenamide, have demonstrated promising efficacy and a manageable safety profile in patients with primary or relapsed refractory lymphoma, either alone or in combination with CHOP or R-CHOP regimens (10–15). Multiple novel immunotherapeutic agents, such as bispecific antibodies and chimeric antigen receptor T-cells, have also shown potential efficacy, introducing novel treatment alternatives for patients who otherwise have no possibility of cure (16).

Nevertheless, there is still a substantial unfulfilled clinical need. Diffuse large B-cell lymphoma is one of the most prevalent lymphomas, and the majority of patients experience improved survival and prognosis with the use of standard first-line treatment regimens based on the R-CHOP. However, treatment failure still occurs in 15% to 25% of individuals, and relapse still occurs in 20% to 30% after first-line therapy (17, 18). In addition, the current understanding of the pathogenesis and biology of T-cell lymphoma is still minimal, rendering it challenging to provide substantive input for its clinical treatment (19). Therefore, more concerted efforts and interdisciplinary studies are urgently needed to join for such a highly heterogeneous malady with complex and multiple treatment strategies and prognostic factors.

In 1924, Otto Warburg, a German biochemist, observed that cancer cells and normal cells follow distinctive patterns of glucose metabolism: even under adequate oxygen conditions, cancer cells prefer glycolysis to the tricarboxylic acid cycle for energy, a phenomenon known as the “Warburg effect” (20). To date, the existence of the Warburg effect has been demonstrated in almost all types of cancer, and the “Warburg effect” has been extensively endorsed as a hallmark feature of cancer (21). The abnormal cancer metabolism effectively promotes critical biological processes such as proliferation, growth, migration and invasion of cancer cells (22–24). Scientists are now striving to prevent or treat cancer by blocking the metabolic hallmark of cancer cells as a means to halt cancer development and progression effectively. Such a strategy is primarily based on the findings that significant metabolic dysfunction exists in almost all types of cancer and that targeting cancer metabolism interferes with almost all hallmarks of cancer (21, 25, 26). In recent years, there has been growing evidence that adaptive metabolic reprogramming is necessary for energy production and intracellular biosynthesis during lymphoma initiation and progression (27). Such an adaptive cancer metabolism has been wielded clinically in lymphoma diagnosis and treatment. For example, PET/CT is available to determine glucose metabolic activity in the most aggressive non-Hodgkin’s lymphoma (NHL), and PET/CT-detected glucose metabolic activity has been demonstrated to correlate positively with the degree of lymphoma aggressiveness (28, 29). In addition, antimetabolic anticancer drugs have become an integral and essential part of clinical oncology drugs such as Methotrexate, Fluorouracil, Cytarabine, etc (30–32). Currently, an increasing number of antitumor drugs based on metabolic interventions are being defined preclinically or are in clinical trials, and targeting metabolic enzymes, metabolites, or their signalling pathways provides spacious boulevards to combat cancer (26, 33–37).

As an interdisciplinary subject of mathematical statistics, computer science and informatics, the application of scientometrics in the biomedical field has suddenly skyrocketed in recent years (38–42). The popularity of scientometric study in biomedical research is not an artistic fashion but a reflection of its tremendous utility in medical research. By rigorously performing mathematical statistics on large amounts of unstructured data, the scientometric study enables (i) profiling the accumulated scientific knowledge in established fields, (ii) decoding the subtle evolution of academic elements and achieving hierarchical clustering of academic elements, (iii) identifying significant research signatures in the field, and (iv) predicting under-explored areas worthy of further study (43–45). As for lymphoma metabolism, a wealth of unstructured data already exists and continues to proliferate every year, rendering it challenging for scholars to read a large amount of unstructured data in a short period, to visually decode the internal structure and connections among the data, to make sense of the evolution of scientific data, and to convincingly identify significant research signatures and future directions worthy of further exploration. The technical merits of scientometrics serve as a robust bridge to quench these challenges.

To the best of our knowledge, despite the apparent technical merits of scientometrics, there is still no scientometric and visualization study regarding the metabolic hallmark of lymphoma. Hence, from a scientometric and visualization perspective, this study aimed to profile current characteristics, uncover evolutionary processes and intrinsic linkages, identify significant research signatures on the lymphoma metabolism, and provide some substantive predictions for this domain.

## 2 Methods

### 2.1 Strict quality control and data source

Several mainstream medical information retrieval and storage databases exist in the biomedical field, such as Web of Science (WOS), Scopus and PubMed. After material investigation and group discussion, the WOS database was finally identified as our data pool for further analysis.

There are many different types of documents in the data pool, such as articles, reviews, case reports, meetings, abstracts, patents, news, letters and corrections, among which articles and reviews are materials that have been rigorously peer-reviewed and contain complete document content. To ensure the reliability and quality of the data, these two were eventually identified as the next step in the data collection. In addition, documents that are too old may need more quality and more validity to be meaningful for the current study; therefore, the last decade was truncated as the time span for this study.

### 2.2 Search strategy

To ensure the completeness and reliability of the search terms, the titles and abstracts of a large number of documents (approximately four hundred) in this field were searched, analyzed, and summarized; in addition, the MeSH database was used for further verification. The topic of this study was “lymphoma metabolism”, which was further divided into “lymphoma” and “metabolism” for search term discovery in order to ensure the comprehensiveness of the subject terms. We first used WOS to search for “lymphoma” and found new synonyms in the title and abstract, while the Mesh database was also used to find additional search terms. The same search term discovery strategy was also used for “metabolism”. Eventually, we found the plural form of “lymphoma” as its supplement and found two deformation words of “metabolism” as its supplement. The search query was “TS=(lymphoma or lymphomas) AND TS=(metabolism or metabolic or metaboly)”. The search duration was from January 1, 2013 to December 31, 2022.

### 2.3 Data collection

The filtering function of the database was used to limit the study time span and article types. In addition, non-English documents were excluded to ensure the stability of the subsequent analysis. From all the above documents, a large amount of lymphoma metabolism-related data was mined, quantified and further stored in Microsoft Excel, including publication year, references, authors, countries, affiliations, journals, global citation number, local citation number, total publication number, total citation number, average citation number, citation number without self-citation, h-index, m-index, g-index and keywords. During the data collection process, one author was identified as the main data collector and the other two authors were assigned to double-check the data further, avoiding mistakes and bias from the data collection.

### 2.4 Metrics interpretation

As standard metrics in scientometrics, publications and citations are used to assess academic performance, with publications indicating academic productivity and citations indicating academic impact and contribution. The global citation number of an article indicates how often the article is cited in the entire WOS database. The number of local citations of an article indicates how often the article is cited in the current dataset, and if an article has a high number of local citations, it means it is an essential article in the field.

The h-index, m-index, and g-index are three hybrid metrics that can comprehensively evaluate academic performance. The h-index means that an academic individual has h articles that have been cited at least h times, thus linking their publication number to their citation number. The h-index is the most commonly used indicator. However, it is not objective for younger or more quality-oriented academics, so the m-index and g-index are also significant indicators. The m-index is an h-index based on academic age, which is equal to the h-index divided by the duration of the academic individual from the first publication to the present. The g-index is the squared number of citations g accumulated by the academic individual who has published at least g articles.

### 2.5 Scientometrics and visualization analysis

Further visualization analysis was made possible by VOSviewer 1.6.18(0), GraphPad Prism 7.0, and R 4.2.2. The R language provides a robust mathematical computing environment for data processing, computation, and graphing, and several R packages (such as “bibliometrix”and “ggplot2”) are utilized to facilitate this study.

VOSviewer, a program based on the Java environment, was used to build and generate visualization networks through co-authorship and co-occurrence analysis (46, 47). In the visualized networks, the nodal values denote the total link strength (TLS), the thickness of the lines indicates the strength of the relationship between two individuals, and the color of the nodes indicates the different clusters or periods. For the multi-network visualized study of authors, countries and affiliations, all elements are first divided into several clusters based on the correlation between different elements, after which the timeline distribution, publication density and citation density between different clusters are further comprehensively compared. Finally, the key clusters and nodes are thus identified (48).

### 2.6 Hierarchical clustering

Hierarchical clustering is one of the most common types of clustering strategies. Firstly, the direct similarity between two individuals in the population is calculated in turn. Specific two close individuals are combined into a new class. The similarity between the new class and other classes is calculated, and the above cycle is repeated continuously, and finally, the population is divided into several optimal clusters. For the visualized clustering study of author keywords based on co-occurrence frequency, the data of several clusters after hierarchical clustering were extracted, after which the specific meanings and weights of keywords were analyzed based on expertise. Thus, the direction of study for specific clusters was determined. Based on the visualized timeline distribution and visualized connection and occurrence densities, various clusters were two-by-two compared, and thus crucial nodes and clusters were identified.

### 2.7 Community map analysis and Walktrap algorithm

Random Walk is one of the most mainstream research strategies in community discovery research, whose central idea is that the initial distribution releases a large number of irregular walkers, and after diffusion, the distribution function of these walkers can be thus derived. The Walktrap algorithm is a framework based on the Random Walk strategy, whose central idea is that nodes in the same community will have more connections, and nodes in different nodes between communities are relatively less connected, so a random direction walker will be trapped inside a community for a longer time. With such a stratified clustering, the Walktrap algorithm can divide the community into a clear stratified structure, thus dividing the density and centrality of different keywords. Density indicates the degree of development, and centrality represents the degree of relevance. The community map further divides all the topic terms into four regions, Motor region, Niche region, Emerging or Declining region and Basic region, by the degree of development and relevance.

### 2.8 Linear regression and statistical analysis

Linear regression is a statistical analysis method used to determine the quantitative relationship between two or more interdependent variables. Through linear regression analysis, we can determine whether there is a dependence between two variables, generate regression curves to predict future trends, calculate the goodness of fit and statistical significance, and determine whether the two variables are statistically significant. The p-value reflects the magnitude of the likelihood of an event occurring, with P < 0.05 being statistically different, P < 0.01 being statistically significantly different, and P < 0.001 being extremely statistically different.

## 3 Results

### 3.1 General overview of the lymphoma metabolism research

**Figure 1:**
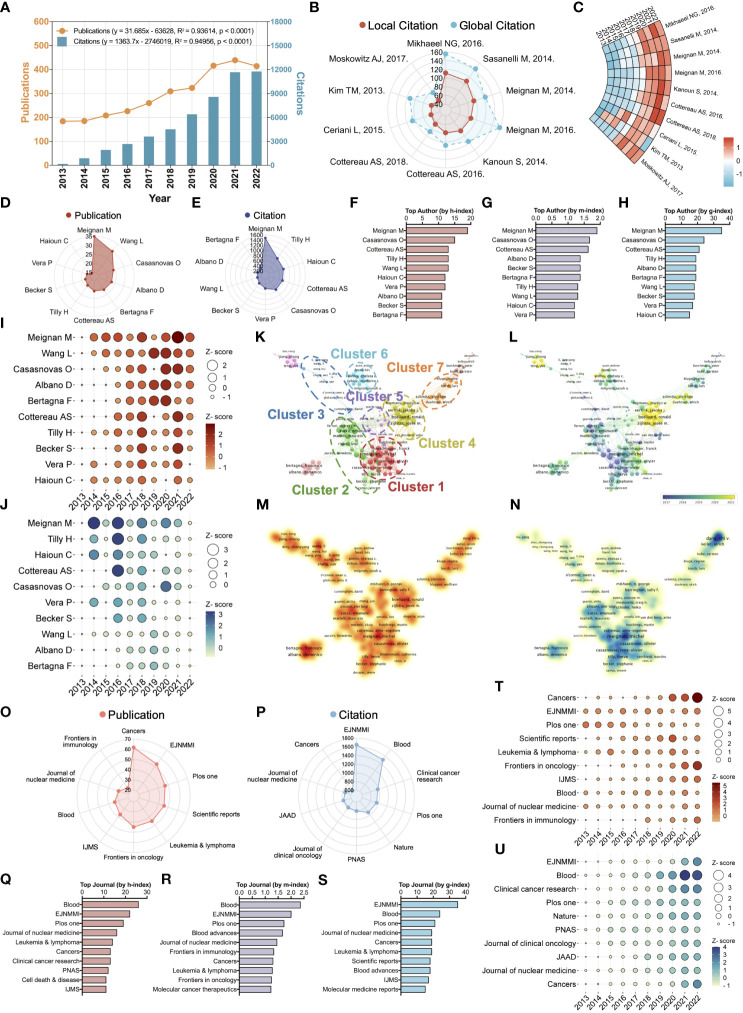
Scientometric and visualized study of the top ten cited papers, productive authors, and journals. **(A)** Timelines and prediction functions of publication (Orange) and citation (Blue) number regarding the lymphoma metabolic research. R^2^ denotes the correlation coefficient. The P-value represents statistical difference. **(B)** A radar chart exhibits the local (Red) and global (Blue) citation rank and the corresponding number of the top ten highest citation papers. Global citation indicates the number of times all documents in the entire WOS database have cited this paper. Local citation indicates the number of times this paper has been cited in the WOS core database. **(C)** A ring heatmap visualized each highest citation paper’s citation number per year. **(D)** Publication rank and the corresponding number of the top ten most productive authors. **(E)** Citation rank and the corresponding number of the top ten most productive authors. **(F)** The h-index rank and the corresponding number of the top ten most productive authors. **(G)** The m-index rank and the corresponding number of the top ten most productive authors. **(H)** The g-index rank and the corresponding number of the top ten most productive authors. **(I)** Annual publication number of the top ten most productive authors. **(J)** Annual citation number of the top ten most productive authors. **(K)** Hierarchical cluster analysis classifies authors into seven clusters based on inter-authorship linkages. Cluster 1: Red; Cluster 2: Green; Cluster 3: Blue; Cluster 4: Yellow; Cluster 5: Purple; Cluster 6: Cyan; Cluster 7: Orange. **(L)** Timeline distribution of author clusters. Purple nodes indicate an earlier appearance while yellow nodes indicate a later appearance. **(M)** Publication density visualization among authors. **(N)** Citation density visualization among authors. **(O)** Publication rank and the corresponding number of the top ten most productive journals. EJNMMI, European Journal of Nuclear Medicine and Molecular Imaging; IJMS, International Journal of Molecular Sciences. **(P)** Citation rank and the corresponding number of the top ten most productive journals. PNAS, Proceedings of the National Academy of Sciences of the United States of America; JAAD, Journal of the American Academy of Dermatology. **(Q)**. The h-index rank and the corresponding number of the top ten most productive journals. **(R)** The m-index rank and the corresponding number of the top ten most productive journals. **(S)** The g-index rank and the corresponding number of the top ten most productive journals. **(T)** Annual publication number of the top ten most productive journals. **(U)** Annual citation number of the top ten most productive journals.

The summary statistics of our data pool are displayed in **Table 1**. For the published documents in this field, their average age is 4.64, and the publication continues to grow at a rate of 9.27% per year. At the level of international co-authorship, the rate of international co-authorship in this field is 26.94%. **Figure 1A** presents an overview of the number of publications and citations per year for lymphoma metabolism from 2013 to 2022. From 2013 to 2022, the annual publication or citation number in this field followed a roughly steady upward trend. Two prediction functions were further developed using linear regression analysis to predict the future trend of publications and citations. The function predicting the publication number per year is “y = 31.685x - 63628” with the correlation coefficient R² = 0.93614. Moreover, the function predicting the citation number per year is “y = 1363.7x - 2746019” with the correlation coefficient R² = 0.94956. The steady increase in publications and citations indicates that lymphoma metabolism has received increasing scholarly attention and is flourishing in the past decade.

**Table 1:** Basic characteristics of the data pool.

Description	Results
Timespan	2013 to 2022
Journals	1035
Documents	2925
article	2403
review	522
References	119853
Authors	18225
Keywords plus	7200
Author’s keywords	6341
Annual growth rate	9.27%
Document average age	4.64
Average citations per document	17.83
Authors of single-authored documents	58
Single-authored documents	60
Co-authors per document	8.39
International co-authorships	26.94%

### 3.2 The core contents of landmark documents based on the highest local citations target metabolic tumor volume and its clinical applications as a pivotal research signature

Global citations indicate the number of times all documents in the entire WOS database have cited this paper. Local citations indicate the number of times this paper has been cited in the WOS core database. Compared to global citations, local citations are a more accurate indicator of reflecting the recognition of core scholars in a particular field, and a paper with high local citations means that it is an important piece of literature in this field. Among the top ten highest local citation papers, two items published by Mikhaeel NG and Sasanelli M, respectively, enjoy more than 100 citations (**Figure 1B**). In the last four years, the top ten highest local citation papers have shown a significant increase in the annual number of citations, with a clear citation stratification compared to the previous period, indicating the growing interest and emergence of the field (**Figure 1C**). The top ten landmark documents based on the highest local citations are displayed in **Table 2**. These documents are published in high-quality journals in this field, such as the European Journal of Nuclear Medicine and Molecular Imaging (4/10), Blood (3/10), Journal of Clinical Oncology (1/10), Clinical Cancer Research (1/10) and Cancer (1/10). Upon deeper analysis, it is astonishing that the core contents of almost all (90%) of these documents point directly to metabolic tumor volume and its clinical applications (such as staging, risk classification, and prognostic prediction). The core content of the other 10% of the documents is total lesion glycolysis, a research signature that is also associated with metabolic tumor volume. These results suggest that metabolic tumor volume and its clinical applications are a pivotal research signature in this field.

**Table 2:** The top ten landmark documents based on the highest local citations.

Document	Title	Journal	IF (2021)	Local Citations	Global Citations
Mikhaeel NG, 2016.	Combination of baseline metabolic tumour volume and early response on PET/CT improves progression-free survival prediction in DLBCL	European Journal of Nuclear Medicine and Molecular Imaging	10.057	112	156
Sasanelli M, 2014.	Pretherapy metabolic tumour volume is an independent predictor of outcome in patients with diffuse large B-cell lymphoma	European Journal of Nuclear Medicine and Molecular Imaging	10.057	109	144
Meignan M, 2014.	Metabolic tumour volumes measured at staging in lymphoma: methodological evaluation on phantom experiments and patients	European Journal of Nuclear Medicine and Molecular Imaging	10.057	95	123
Meignan M, 2016.	Baseline Metabolic Tumor Volume Predicts Outcome in High-Tumor-Burden Follicular Lymphoma: A Pooled Analysis of Three Multicenter Studies	Journal of Clinical Oncology	50.717	88	158
Kanoun S, 2014.	Baseline metabolic tumour volume is an independent prognostic factor in Hodgkin lymphoma	European Journal of Nuclear Medicine and Molecular Imaging	10.057	86	116
Cottereau AS, 2016.	Molecular Profile and FDG-PET/CT Total Metabolic Tumor Volume Improve Risk Classification at Diagnosis for Patients with Diffuse Large B-Cell Lymphoma	Clinical Cancer Research	13.801	79	108
Cottereau AS, 2018.	Prognostic value of baseline metabolic tumor volume in early-stage Hodgkin lymphoma in the standard arm of the H10 trial	Blood	25.476	67	88
Ceriani L, 2015.	Utility of baseline 18FDG-PET/CT functional parameters in defining prognosis of primary mediastinal (thymic) large B-cell lymphoma	Blood	25.476	62	107
Kim TM, 2013.	Total lesion glycolysis in positron emission tomography is a better predictor of outcome than the International Prognostic Index for patients with diffuse large B cell lymphoma	Cancer	6.921	61	113
Moskowitz AJ, 2017.	Prognostic significance of baseline metabolic tumor volume in relapsed and refractory Hodgkin lymphoma	Blood	25.476	54	76

### 3.3 Scientometric and visualized study of the top ten productive authors

Identifying outstanding scholars in a field will assist us in tracking cutting-edge advances in a given field. After a comprehensive analysis, the two scholars, Meignan Michel and Casasnovas Olivier, consistently present in the top five of all metrics (**Figures 1D-H**). The evolution of the annual publication of the top ten most productive authors indicates that Meignan Michel and Casasnovas Olivier have maintained a high output for the last three years (**Figure 1I**). The evolution of the annual citation of the top ten most productive authors shows that the annual citation of Meignan Michel reached its highest in 2014, while Casasnovas Olivier’s reached its highest in 2020 (**Figure 1J**). After a hierarchical cluster analysis, the 18,225 authors were classified into several clusters based on inter-authorship linkages (**Figure 1K**). Cluster 1: Red; Cluster 2: Green; Cluster 3: Blue; Cluster 4: Yellow; Cluster 5: Purple; Cluster 6: Cyan; Cluster 7: Orange. The two scholars mentioned above are located in cluster 1 among these seven clusters. Among the seven mentioned clusters, Meignan Michel (TLS = 126), Zucca Emanuele (TLS = 74), Cunningham David (TLS = 18), Boellaard Ronald (TLS = 103), Buvat Irene (TLS = 22), Pinnix Chelsea c. (TLS = 39) and Schmitz Christine (TLS = 39) are the pivotal nodes of the collaborative network. The timeline distribution of author clusters indicates that Cluster 2 emerged early and developed over a long period, while Cluster 4 emerged late (**Figure 1L**). Publication density visualization among authors indicates that clusters 1 and 4 have higher publications than others (**Figure 1M**). Citation density visualization among authors indicates cluster 1 has higher citations than others (**Figure 1N**). The results show that Meignan Michel, Casasnovas Olivier and Cluster 1 have a comprehensive academic performance that could be followed in subsequent studies to keep abreast of the frontier progress in the field.

### 3.4 Scientometric and visualized study of the top ten productive journals

Discovering quality journals and getting manuscripts published in them will allow our work to reach a broader readership. After a comprehensive analysis, the journal European Journal of Nuclear Medicine and Molecular Imaging (EJNMMI) consistently presents in the top three of all metrics (**Figures 1O-S**). In addition, despite the small number of articles, other metrics of the journal Blood are in the top three. The evolution of the publication volume per year of the top ten most productive journals indicates that EJNMMI and Blood maintain a steady volume of publications each year (**Figure 1T**). The evolution of the citation volume per year of the top ten most productive journals indicates that EJNMMI and Blood have an increasing number of annual citations (**Figure 1U**). These results suggest that EJNMMI and Blood are quality journals in the field, suitable not only for follow-up studies but also for the publication of subsequent research work.

### 3.5 The comprehensive performance and development evolution of the top ten most productive countries and affiliations

**Figure 2:**
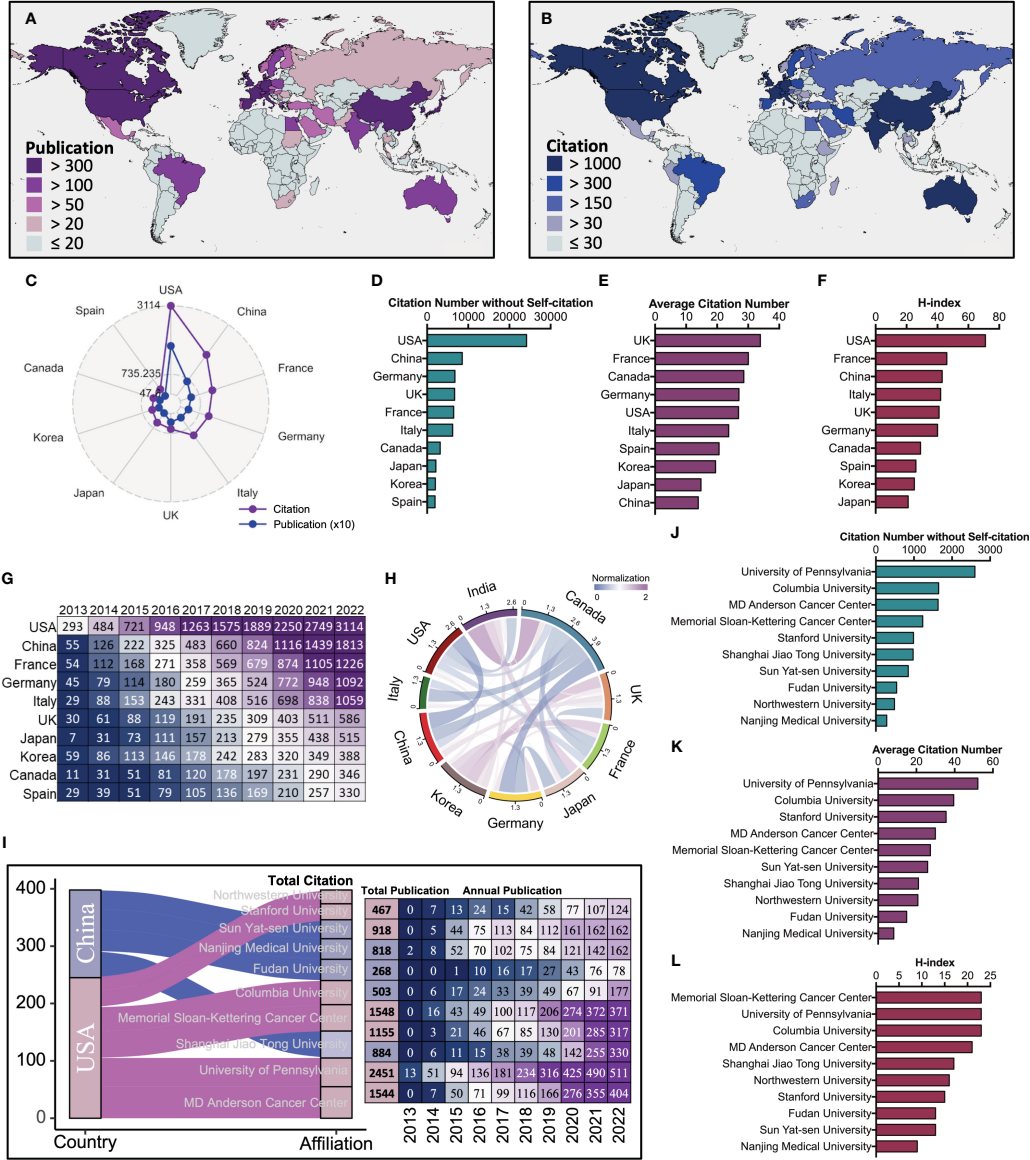
The comprehensive performance and development evolution of the top ten most productive countries and affiliations. **(A)** Geographical distribution of publication volume by countries. **(B)** Geographical distribution of citation volume by countries. **(C)** Publication (Blue) and citation (Purple) number of the top ten most productive countries. **(D)** Citation number without self-citation rank and the corresponding number of the top ten most productive countries. **(E)** Average citation rank and the corresponding number of the top ten most productive countries. **(F)** The h-index rank and the corresponding number of the top ten most productive countries. **(G)** Cumulative citation rank and the corresponding number of the top ten most productive countries. **(H)** The Circos diagram displays the network of partnerships between different countries. **(I)** Left, the Sankey diagram displays country sources and total citation of the top ten most productive affiliations. Right, the total publication and annual publication for the corresponding affiliations. **(J)** Citation number without self-citation and rank of the top ten most productive affiliations. **(K)** Average citation rank and the corresponding number of the top ten most productive affiliations. **(L)** The h-index rank and the corresponding number of the top ten most productive affiliations.

The global distribution of publication and citation volume shows a high consistency in the trend of these two metrics across countries, where the USA, China and France are firmly in the top three (**Figures 2A-C**). In addition, the USA, UK, and France are consistently within the top five for the other metrics (citation number without self-citation, average citation number, h-index) (**Figures 2D-F**). Cumulative citation rank and the corresponding volume of the top ten most productive countries show that the USA has been at the top of the list and is way ahead of other countries (**Figure 2G**). However, China has been steadily increasing in annual citation volume and overtaking the USA in 2022. The network diagram of partnerships between different countries shows close and complex cooperation between different countries in this field (**Figure 2H**). The result of the total citations of the top ten most productive affiliations shows that MD Anderson Cancer Center and University of Pennsylvania from the USA and Shanghai Jiao Tong University from China are in the top three, and further analysis of annual publications shows that all three affiliations also have steadily increasing annual publications (**Figure 2I**). In addition, the University of Pennsylvania from the USA is consistently within the top three for the other metrics (citation number without self-citation, average citation number, h-index) (**Figures 2J-L**). These results indicate that the USA and its affiliation University of Pennsylvania have a comprehensive academic performance that warrants follow-up for outstanding progress and collaboration in the field and that China and its affiliations are equally noteworthy as an emerging entity that is gradually developing.

### 3.6 Multi-network visualized study of countries and affiliations

**Figure 3:**
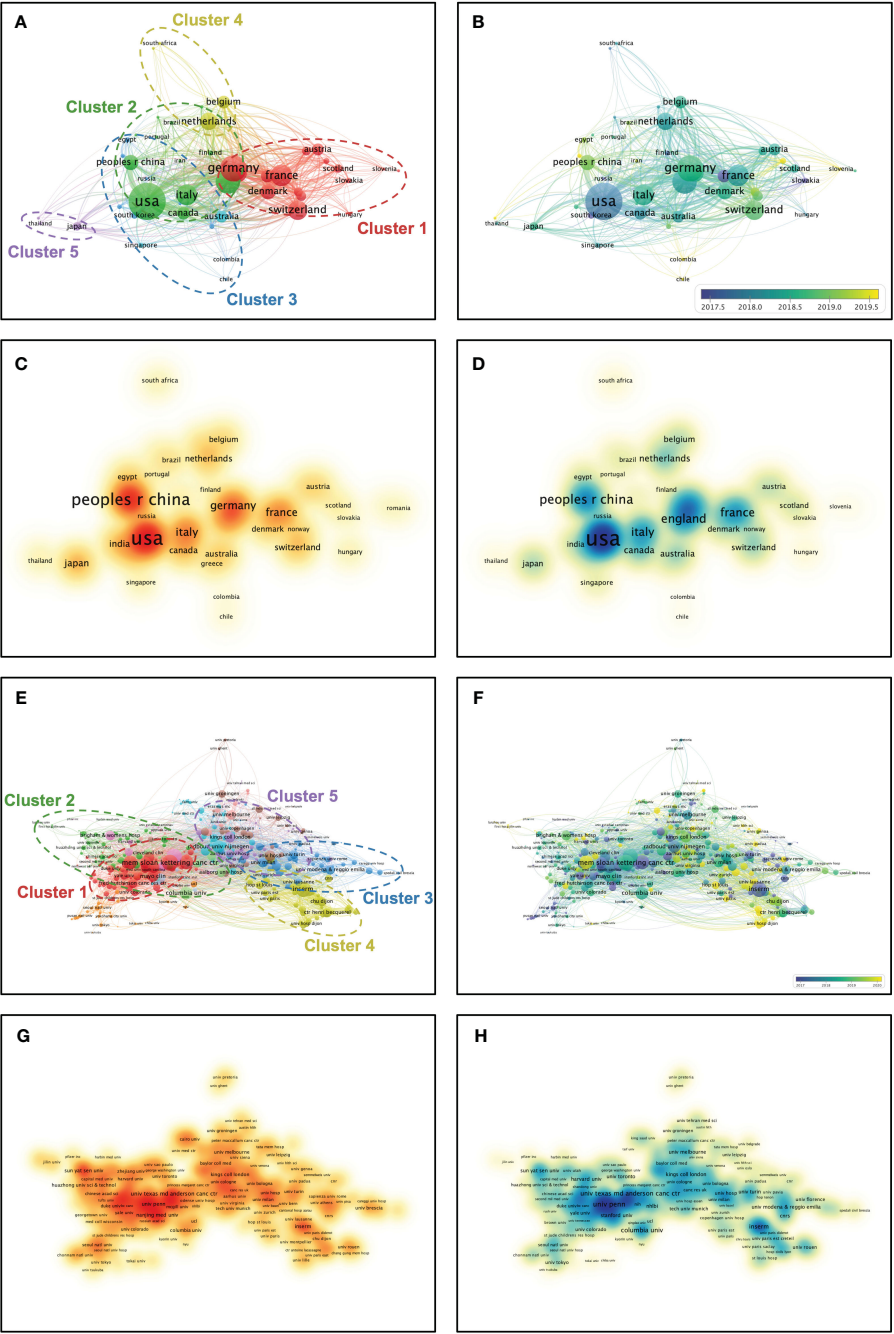
Multi-network visualized study of countries and affiliations. **(A)** Hierarchical clustering analysis classifies the countries into five clusters based on the total linkage strength. Cluster 1: Red; Cluster 2: Green; Cluster 3: Blue; Cluster 4: Yellow; Cluster 5: Purple. **(B)** Timeline distribution of cluster analysis of the countries. Purple nodes indicate an earlier appearance while yellow nodes indicate a later appearance. **(C)** Visualized publication density among the countries. **(D)** Visualized citation density among the countries. **(E)** Hierarchical cluster analysis classifies the affiliations into five clusters based on the total linkage strength. Cluster 1: Red; Cluster 2: Green; Cluster 3: Blue; Cluster 4: Yellow; Cluster 5: Purple. **(F)** Timeline distribution of cluster analysis of the affiliations. Purple nodes indicate an earlier appearance while yellow nodes indicate a later appearance. **(G)** Visualized publication density among the affiliations. **(H)** Visualized citation density among the affiliations.

After a hierarchical cluster analysis, the 51 countries were classified into several clusters based on inter-country linkages (**Figure 3A**). Cluster 1: Red; Cluster 2: Green; Cluster 3: Blue; Cluster 4: Yellow; Cluster 5: Purple. The two countries mentioned above (USA and China) are located in cluster 2 among these five clusters. Among the five mentioned clusters, Germany (TLS = 417), USA (TLS = 673), Korea (TLS = 65), Netherlands (TLS = 240), and Japan (TLS = 89) are the pivotal nodes of the collaborative network. Timeline distribution of country clusters indicates that Cluster 2 emerged early and developed over a long period, while Cluster 1 emerged late (**Figure 3B**). Publication density visualization among countries indicates that cluster 2 has higher publications than others (**Figure 3C**). Citation density visualization among countries indicates that cluster 2 has higher citations than others (**Figure 3D**). After a hierarchical cluster analysis, the 404 affiliations were classified into several clusters based on inter-affiliation linkages (**Figure 3E**). Cluster 1: Red; Cluster 2: Green; Cluster 3: Blue; Cluster 4: Yellow; Cluster 5: Purple. The affiliation University of Pennsylvania mentioned above is located in cluster 1 among these five clusters. Among the five mentioned clusters, Memorial Sloan-Kettering Cancer Center (TLS = 129), University of Toronto (TLS = 43), University of Milan (TLS = 64), Inserm (TLS = 118), and King’s College London (TLS = 70) are the pivotal nodes of the collaborative network. Timeline distribution of affiliation clusters indicates that Cluster 1 emerged early and developed over a long period, while Cluster 4 emerged late (**Figure 3F**). Publication density visualization among affiliations indicates that cluster 1 has higher publications than others (**Figure 3G**). Citation density visualization among affiliations indicates that cluster 1 has higher citations than others (**Figure 3H**). The results show that Cluster 2 of country clusters and Cluster 1 of affiliation clusters have a comprehensive academic performance that could be followed in subsequent studies to keep abreast of the frontier progress in the field.

### 3.7 Multi-network visualized study of research hotspots of the lymphoma metabolism

**Figure 4:**
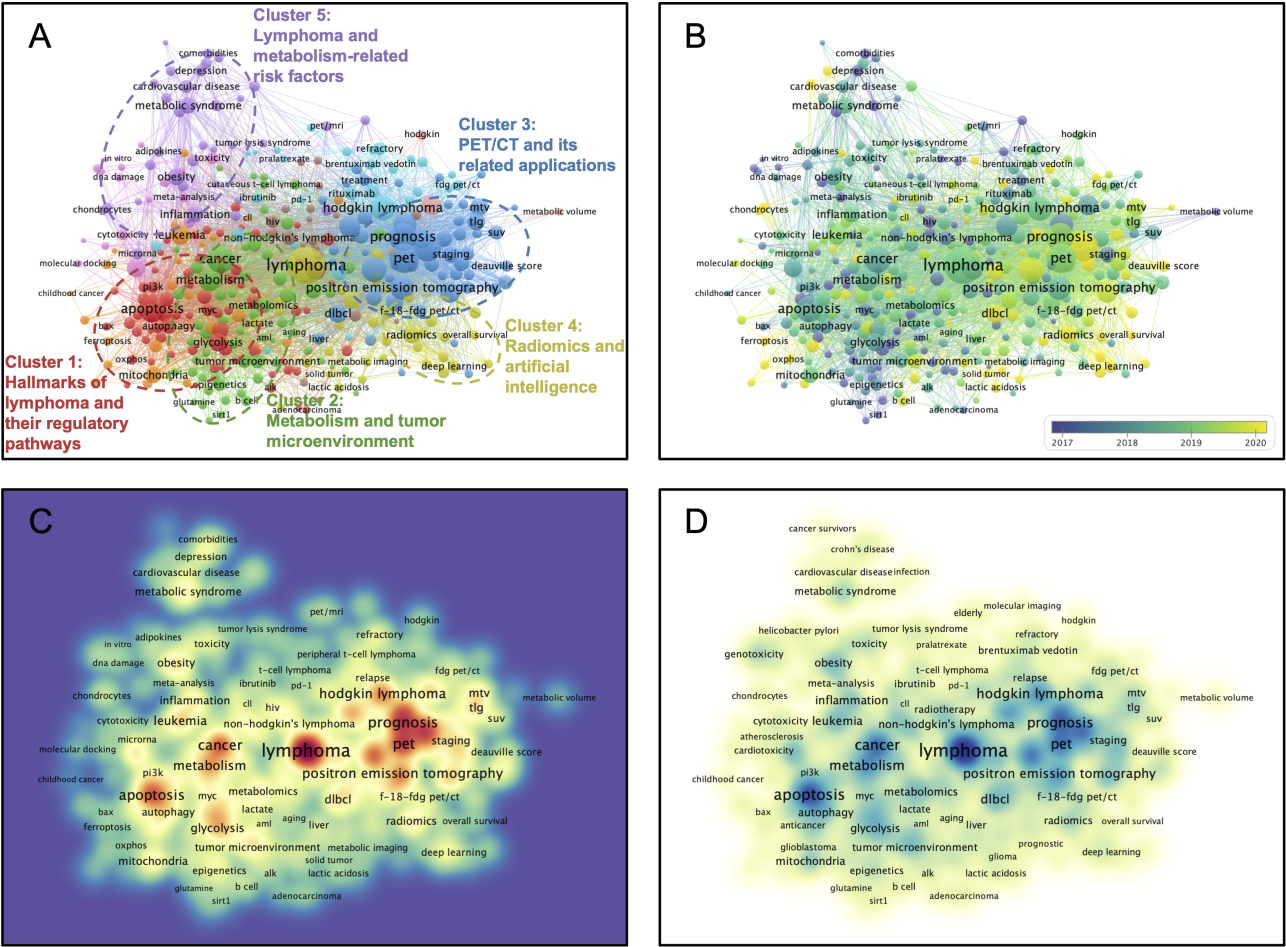
Multi-network visualized study of research hotspots of the lymphoma metabolism. **(A)** Visualized clustering study of author keywords based on co-occurrence frequency. The nodal values denote the frequency of co-occurrence. Cluster 1 (Red): Hallmarks of lymphoma and their regulatory pathways. Cluster 2 (Green): Metabolism and tumor microenvironment. Cluster 3 (Blue): PET/CT and its related applications. Cluster 4 (Yellow): Radiomics and artificial intelligence. Cluster 5 (Purple): Lymphoma and metabolism-related risk factors. **(B)** Timeline distribution of the author keywords. Purple nodes indicate an earlier appearance while yellow nodes indicate a later appearance. **(C)** Visualized connection density of keywords with other keywords. **(D)** Visualized occurrence density of keywords.

A hierarchical cluster strategy was applied for visualized clustering study of author keywords based on co-occurrence frequency (**Figure 4A**). All author keywords were limited to at least five occurrences, and 347 items were eventually generated and further utilized in the clustering study. These items were classified into ten clusters, with the first five clusters being further defined and boxed out with circles (Cluster 1: Red, Hallmarks of lymphoma and their regulatory pathways study; Cluster 2: Green, Metabolism and tumor microenvironment study; Cluster 3: Blue, PET/CT and its related applications study; Cluster 4: Yellow, Radiomics and artificial intelligence study; Cluster 5: Purple, Lymphoma and metabolism-related risk factors study). After excluding some confounding items, apoptosis (TLS = 246), glucose metabolism (TLS = 59), pet/ct (TLS = 325), radiomics (TLS = 82), and obesity (TLS = 82) are identified as the critical nodes of the collaboration network among these five clusters. In addition, two keywords, pet/ct (TLS = 325) and prognosis (TLS = 305), were determined to be the top two highest in total link strength in all keywords after excluding some confounding items. Timeline distribution of keyword clusters indicates that Cluster 2 emerged early and developed over a long period, while Cluster 4 emerged late (**Figure 4B**). Connection density visualization among keywords indicates that cluster 3 connects more closely with keywords than others (**Figure 4C**). Occurrence density visualization among keywords indicates clusters 1 and 3 have higher occurrences than others (**Figure 4D**). The keywords “pet/ct”, “prognosis”, and “apoptosis” have a high density between connection and occurrence.

### 3.8 The burst, evolution, and statistical analysis of research signatures of the lymphoma metabolism

**Figure 5:**
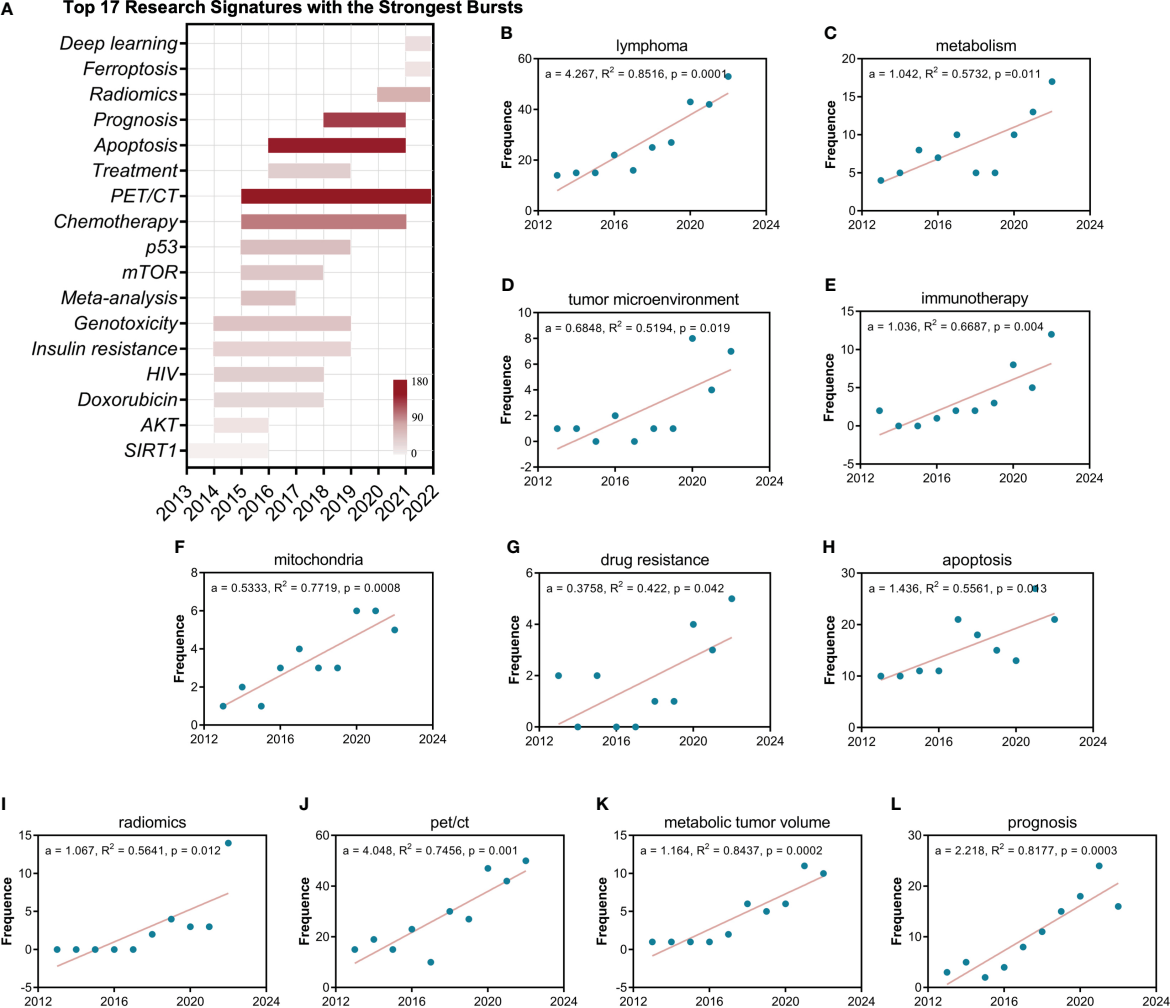
The burst, evolution, and statistical analysis of research signatures of the lymphoma metabolism. **(A)** The burst and evolution of the top seventeen research signatures with the strongest bursts. The depth of red represents the strength of the burst. **(B-L)** Linear regression analysis of the annual publication frequency of research signatures. The a represents the slope. R^2^ denotes the correlation coefficient. The P-value represents statistical difference.

The burst analysis shows that among these 17 research signatures, Apoptosis, PET/CT and Prognosis have the strongest burst (**Figure 5A**). Furthermore, the evolutionary analysis shows that the early research in this field focused on some star molecules, such as SIRT1, AKT, mTOR and p53, and the recent research gradually shifted to Ferroptosis, Radiomics and Deep learning. All linear regression analyses (**Figures 5B-L**) showed statistically significant differences (p > 0.05), especially for the search terms lymphoma and metabolism, indicating the plausibility of this analysis strategy (**Figures 5B, C**). Research signatures pet/ct (**Figure 5J**) and prognosis (**Figure 5L**) demonstrate outstanding burst trends (a > 2), and research signatures apoptosis (**Figure 5H**), metabolic tumor volume (**Figure 5K**), radiomics (**Figure 5I**), and immunotherapy (**Figure 5E**) display promising burst trends (a > 1).

### 3.9 The potentiality prediction of research signatures of the lymphoma metabolism

**Figure 6:**
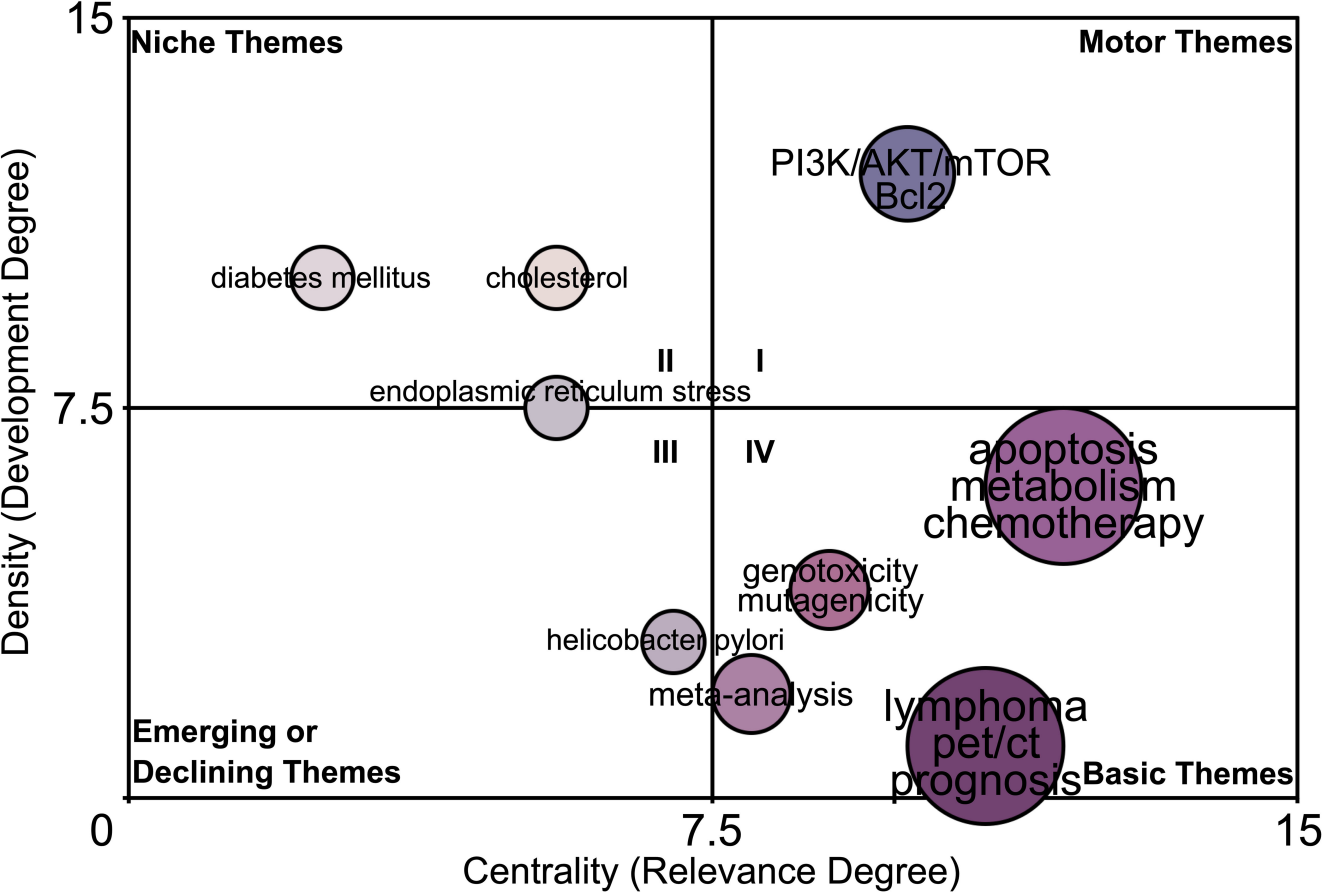
The potentiality prediction of research signatures of the lymphoma metabolism. The “Walktrap” algorithm based on the Random Walk strategy was utilized to perform community discovery analysis on 5,000 author keywords. The value of the x-axis indicates the relevance (importance) of the research signatures to the field, while the value of the y-axis indicates the degree of development of the research signatures in the field. Keywords “lymphoma, pet/ct, prognosis”, “apoptosis, metabolism, chemotherapy”, “meta-analysis”, and “genotoxicity, mutagenicity” in Community IV (Crucial but still under-explored signatures).

The “Walktrap” algorithm was utilized to perform community discovery analysis on 5,000 author keywords. (**Figure 6**). In the motor theme community (quadrant I), “PI3K/AKT/mTOR, Bcl2” are defined as important and well-developed. In the niche theme community (quadrant II), “diabetes mellitus”, “cholesterol”, and “endoplasmic reticulum stress” are defined as well-developed but unimportant to the current research. In the emerging or declining theme community (quadrant III), “helicobacter pylori” is defined as poor-developed and marginal. More interestingly, the community map shows that the “lymphoma, pet/ct, prognosis”, “apoptosis, metabolism, chemotherapy”, “genotoxicity, mutagenicity”, and “meta-analysis” are crucial to the metabolic hallmark of lymphoma but still under-explored, illustrating the research potentiality of these research signatures in the field of the metabolic hallmark of lymphoma.

## 4 Discussion

To the best of our knowledge, this study, for the first time, revealed a global scientific landscape of lymphoma metabolism-related research published in the last decade through scientometric metrics and information visualization techniques, including the temporal and spatial distribution of the documents and their contributions, and the authors, journals, countries, affiliations, and their evolution and network connections. Through hierarchical analysis, this study established several key research signature clusters and their temporal evolution and network connections. Further burst analysis and linear regression analysis identified several significant research signatures. More interestingly, by implementing the Walktrap algorithm, this study predicted several under-explored areas that are essential for the lymphoma metabolism but still need further study.

The publication and citation volume of articles reflects the development of a certain research field over the years. Over the past ten years, publication and citation volume on lymphoma metabolism have consistently shown a significant increasing trend without much fluctuation. Linear regression analysis developed two functions predicting the publication and citation number per year (Publication: y = 31.685x - 63628, R² = 0.93614, p < 0.0001; Citation: y = 1363.7x - 2746019, R² = 0.94956, p < 0.0001). Based on the prediction functions, we further predict 598 publications and 18201 citations in this field in 2027. These results suggest that the metabolic hallmark of lymphoma has received increasing attention and has been burgeoning over the past decade.

After an in-depth analysis of landmark documents based on the highest local citations, it is astonishing that the core contents of almost all (90%) of these documents point directly to metabolic tumor volume and its clinical applications (such as staging, risk classification, and prognostic prediction). The core content of the other 10% of the documents is total lesion glycolysis, a research signature that is also associated with metabolic tumor volume. These results suggest that metabolic tumor volume and its clinical applications are a pivotal research signature in this field. In addition, these documents are all published in high-quality journals in this field, such as the European Journal of Nuclear Medicine and Molecular Imaging (4/10, IF = 10.057), Blood (3/10, IF = 25.476), Journal of Clinical Oncology (1/10, IF = 50.717), Clinical Cancer Research (1/10, IF = 13.801) and Cancer (1/10, IF = 6.921), which is further evidence of the reliability of the above conclusion. Metabolic tumor volume, representing the volume of tumor tissue with high metabolic activity, is most commonly measured by a fixed threshold method. By setting the threshold, specific software will segment the lesion in the tumor region, outline the region of interest, and automatically calculate the tumor volume (49, 50). Nowadays, metabolic tumor volume has been applied generally in the diagnosis, staging, efficacy assessment and prognosis prediction of cancer in clinical practice. Despite already being a consensus, it is of interest that this study, for the first time from a scientometric perspective, reconfirms that metabolic tumor volume and its clinical applications are a pivotal research signature in this field.

The results of visualized multi-network of research hotspots indicated that in basic research, the current hotspots on the lymphoma metabolic hallmark focus on hallmarks of lymphoma and their regulatory pathways (Cluster 1). These hallmarks of lymphoma contain apoptosis (TLS = 246, Occurrence = 158), proliferation (TLS = 71, Occurrence = 24), autophagy (TLS = 59, Occurrence = 32), and drug resistance (TLS = 49, Occurrence = 19). And the regulatory pathways of these hallmarks contain ros (TLS = 102, Occurrence = 42), glycolysis (TLS = 97, Occurrence = 34), c-myc (TLS = 57, Occurrence = 25), bcl-2 (TLS = 50, Occurrence = 25), p53 (TLS = 44, Occurrence = 20), mtor (TLS = 36, Occurrence = 15), hypoxia (TLS = 32, Occurrence = 19), pi3k (TLS = 31, Occurrence = 10), ampk (TLS = 19, Occurrence = 6), alk (TLS = 18, Occurrence = 11), bax (TLS = 13, Occurrence = 5), and p-glycoprotein (TLS = 9, Occurrence = 5). Moreover, the clustering results provide some strategies to intervene in these hallmarks, such as curcumin (TLS = 24, Occurrence = 10), methotrexate (TLS = 19, Occurrence = 16), crizotinib (TLS = 16, Occurrence = 58), and quercetin (TLS = 11, Occurrence = 5). The above results reveal a wealth of potential targets and drugs for treating lymphoma and merit further investigation for eventual translation into the clinic.

Moreover, in clinical research, the current hotspots on the lymphoma metabolic hallmark focus on PET/CT and its related applications (Cluster 3). The metabolic parameters of PET/CT include metabolic tumor volume (TLS = 266, Occurrence = 84), total lesion glycolysis (TLS = 153, Occurrence = 42), maximum standardized uptake value (TLS = 55, Occurrence = 22), standardized uptake value (TLS = 65, Occurrence = 21), and total metabolic tumor volume (TLS = 39, Occurrence = 15). Moreover, these metabolic parameters are available for prognosis (TLS = 305, Occurrence = 108) or survival (TLS = 53, Occurrence = 20) prediction, staging (TLS = 66, Occurrence = 21), treatment response assessment (TLS = 35, Occurrence = 14), and diagnosis (TLS = 31, Occurrence = 14). The above findings reveal the importance of PET/CT metabolic parameters and their clinical application, and how to use these metabolic parameters or whether new imaging parameters could be introduced to further optimize the value of PET/CT for clinical application is an essential question for current research. Recent emerging radiomics and deep learning algorithms provide substantial strategies to fully utilize the vast amount of image information in PET/CT to improve its clinical application and to identify novel image biomarkers to command its clinical use. The recent demonstration by Eertink et al. that baseline PET radiomics is superior to traditional IPI risk scores in predicting outcome in diffuse large B-cell lymphoma provides a wonderful example (51).

The results of the timeline distribution of keyword clusters indicate that radiomics and artificial intelligence is a relatively new research cluster. Radiomics is a technology for high-throughput mining quantitative image features from standardized medical imaging images, and further feature selection and model construction ensure the feasibility of diagnosis, classification, and prediction in cancer clinical scenarios (52). Radiomics features (e.g., intensity, shape, texture, or wavelets) provide a wealth of quantitative information about the cancer hallmarks and the tumor microenvironment that is distinct and complementary to other relevant data, including clinically acquired, treatment-related, or genomic data (53). When the radiomics data are fused with other relevant data and then correlated with outcome data, an accurate and reliable clinical decision support system can be generated (54, 55). Earlier, radiomics features were mainly limited to those obtained using pyradiomcs (56), while researchers later found that using deep learning techniques could obtain higher throughput signatures and eventually improve model performance (57). Previous investigations were confined to the tumor region but later found that features from the peri-tumor region were equally valuable, which was a preliminary reflection of the regional heterogeneity of the tumor, in fact (58–60). Recently, unsupervised clustering strategies based on voxels have been introduced to further divide tumor into multiple distinct regions to characterize tumor heterogeneity and even tumor microenvironment, termed tumor habitat radiomics studies (61–65). Moreover, a deep-learning-based 3D super-resolution technique seems to improve the performance to characterize tumor heterogeneity or microenvironment (66, 67). These related attempts are also being executed in our cancer center. In conclusion, in the era of individualized cancer precision therapy, radiomics and artificial intelligence studies are undoubtedly becoming increasingly important, and more endeavors deserve.

Research signature burst analysis, and statistical analysis further confirmed many of the above explorations. The result of multi-network visualized study of field hotspots that the research signatures “pet/ct”, “prognosis”, and “apoptosis” have a high density between connection and occurrence is consistent with the result of burst analysis of research signatures that Apoptosis, PET/CT and Prognosis have the strongest burst and also further obtained the statistical confirmation from the linear regression analysis. The result of the core contents analysis of landmark documents that metabolic tumor volume and its clinical applications are a pivotal research signature in this field further obtained the statistical confirmation from the linear regression analysis. The result of the timeline distribution of keyword clusters that radiomics and artificial intelligence is a relatively new research cluster is consistent with the result of burst analysis of research signatures that Radiomics and Deep learning burst from 2020 and also further obtained the statistical confirmation from the linear regression analysis. Indeed, the results of many analyses from different perspectives in this study corroborate each other and are difficult to enumerate exhaustively.

The community map showed that in the motor theme community (quadrant I), “PI3K/AKT/mTOR, Bcl2” are defined as important and well-developed and in the emerging or declining theme community (quadrant III), “helicobacter pylori” is defined as poor-developed and marginal. Indeed, numerous existing studies have confirmed these findings. The PI3K/AKT/mTOR pathway is one of the most important intracellular signalling pathways, responsible for regulating biological processes such as cancer cell growth, proliferation and migration, which is closely related to cancer initiation and development and has become one of the popular pathways for researchers to develop cancer targeted drugs (68–70). Bcl2 is a star protein that inhibits apoptosis, and targeting the PI3K/AKT/mTOR pathway causes downregulation of Bcl2 protein expression, ultimately leading to lymphoma cell death (71–75). Furthermore, in clinical practice, helicobacter pylori is indeed a relatively marginal factor in all lymphoma subtypes and only associates with gastric mucosa-associated lymphoid tissue lymphoma (76, 77). These results confirmed the reliability of the Walktrap algorithm based on the Random Walk strategy. More interestingly, the community map shows that the “lymphoma, pet/ct, prognosis”, “apoptosis, metabolism, chemotherapy”, “genotoxicity, mutagenicity”, and “meta-analysis” are crucial to the metabolic hallmark of lymphoma but still under-explored, illustrating the research potentiality of these research signatures in the field of the metabolic hallmark of lymphoma.

As an interdisciplinary subject of mathematical statistics, computer science and informatics, the application of scientometrics in the biomedical field has suddenly skyrocketed in recent years. However, the vast majority of current scientometric studies focus on the discoveries of authors, journals, countries and affiliations and superficial subject term analysis, which need to be more flavorful. This study introduces linear regression and statistical analysis to provide further solid statistical guarantees for the reliability of the discovered research signatures. Moreover, this study introduces the Walktrap algorithm based on the Random Walk strategy to predict potential research hotspots that are important to this field but still need to be developed. In addition, many current studies ignore that the semantic analysis provided by VOSviewer or CiteSpace, two mainstream tools for scientometric research, is based on an unsupervised algorithm, and there are many synonymously different words in the final results, which significantly affect the accuracy of the results. This study, therefore, utilizes a post-processing strategy to eliminate the confounding words obtained by the unsupervised algorithm and to merge the synonyms obtained by the unsupervised algorithm with the metrics. In a nutshell, this study contributes a research paradigm for scientometrics and visualization studies and is expected to provide reference implications for subsequent studies.

Several limitations still exist in the present study. First, in order to implement strict quality control and ensure reliable findings, only data from the WOSCC database were accepted in this study, and some data from other databases (e.g., Scopus and PubMed) may have been omitted. However, there is a large amount of duplicate data between different databases. Although some of the duplicate data in these databases can be excluded through data cleaning, the simultaneous inclusion of different databases will inevitably cause the issue of a double weighting of data, eventually interfering with the results of the study. Moreover, the data in other databases but not in WOS may be of poor quality, which will likewise interfere with the final results and conclusions. In addition, to further ensure the quality of the data from WOS, the data pool was restricted to the WOS core database (WOSCC). In conclusion, the large amount of high-quality data in the WOSCC database is sufficient to represent significant discoveries and advances in this field and to ensure the reliability of the conclusions of this study. Second, at the time of performing this study, the data from 2023 were incomplete, so we negotiated and determined to remove these incomplete data, and some conclusions may be slightly changed if the complete data from 2023 are included in subsequent studies. Third, this study chose from 2013 to 2022 as the time frame while a more extended time frame maybe yield more valuable insights. The value of a more extended time frame includes but is not limited to: (1) more robust performance and higher accuracy for all linear regression curves in this article; (2) wider temporal magnitude and more information for the temporal evolution of the academic elements; (3) more layers and more information for hierarchical clustering; (4) broader accumulation amount and more accurate profile for the cross-sectional situation of academic elements. Finally, due to space limitations, there is still a wealth of potentially valuable information in the visualization results that have not been explored in-depth and is expected to be further explored in subsequent studies.

## 5 Conclusion

In conclusion, from a scientometric and visualization perspective, this study profiled current characteristics, uncovered evolutionary processes and intrinsic linkages, identified significant research signatures on lymphoma metabolism, and provided some substantive predictions for this domain.

## Data availability statement

Publicly available datasets were analyzed in this study. This data can be found here: https://www.webofscience.com/wos/alldb/basic-search.

## Author contributions

SG: Conceptualization, Investigation, Methodology, Validation, Writing – original draft. DP: Investigation, Methodology, Validation, Writing – original draft. NS: Investigation, Validation, Writing – original draft. MH: Investigation, Methodology, Validation, Writing – original draft. ZZ: Investigation, Methodology, Validation, Writing – original draft. WH: Project administration, Supervision, Writing – review & editing, Conceptualization. XT: Conceptualization, Project administration, Supervision, Writing – review & editing.
